# Music feels like moods feel

**DOI:** 10.3389/fpsyg.2014.00327

**Published:** 2014-04-16

**Authors:** Kris Goffin

**Affiliations:** Department of Philosophy and Moral Sciences, Ghent UniversityGhent, Belgium

**Keywords:** music and emotion, mood, aesthetic emotion, philosophy of music, vitality affects, emotional expressivity, emotional arousal

## Abstract

While it is widely accepted that music evokes moods, there is disagreement over whether music-induced moods are relevant to the aesthetic appreciation of music as such. The arguments against the aesthetic relevance of music-induced moods are: (1) moods cannot be intentionally directed at the music and (2) music-induced moods are highly subjective experiences and are therefore a kind of mind-wandering. This paper presents a novel account of musical moods that avoids these objections. It is correct to say that a listener’s entire mood is not relevant to the aesthetic appreciation of music. However, the experience of mood consists of having different feelings. Music induces feelings that are intentionally directed at the music and clusters of these feelings can be recognized as typical of a specific mood. Therefore, mood-feelings are relevant to the aesthetic appreciation of music.

## INTRODUCTION

In everyday life, moods and music seem to be linked. You might listen to sad music after having gone through a breakup or to energetic songs to improve your mood instead. Some philosophers maintain that music evokes moods and that they are important to the appreciation of music, while others argue that even if music induces moods, they are irrelevant to the aesthetic experience. By “the aesthetic experience of music” I mean the listener’s evaluative experience of the music which constitutes the listener’s belief that the music has aesthetic value. This is broader than just the enjoyment of music. There is disagreement on what is to be included in this appreciative experience. The arguments against the aesthetic relevance of music-induced moods are: (1) moods do not have intentional objects and therefore cannot be directed at the music (Zangwill, 2004) and (2) music-induced moods are a kind of mind-wandering, as they are personal and vary from listener to listener (Kivy, 2006, 2007). I will present a new account which claims that moods are indeed relevant to the aesthetic experience, but avoids these criticisms. The relation between feeling and mood has been overlooked in the debate on music and mood: musical moods should be considered clusters of feelings and music feels the way moods feel.

## THE VALUE OF MUSICAL MOODS

The following arguments have been given in favor of the relevance of moods for the aesthetic experience of music. The debate mostly deals with absolute or instrumental music, but for the sake of brevity I will use the word “music.” Drawing on empirical research (for example Krumhansl, 1997; Juslin and Sloboda, 2001), Robinson (2005) ascribes a specific role to moods in appreciating music. The mood the music evokes, can help the listener to focus on the expressive qualities of the music. Since moods create bias, moods can affect, for instance, the way we perceive. If we are in a happy mood, we are more inclined to think that the faces we encounter look happy (Niedenthal et al., 2001). In the same way Robinson wants to argue that music elicits a mood which will help to focus the listener’s attention more on the specific expressive qualities of the music. For instance, the languorous mood evoked by listening to Debussy’s “*Pré*lude à l’après-midi d’un faune” plays an important role in appreciating the languorous quality of the music.

Carroll (2003), Carroll and Moore (2007) also defends the importance of moods in appreciating the expressiveness of music, arguing that music cannot express only an emotion but can also express a mood. The appreciation of *L’aprè*s-midi largely consist in recognizing it as expressive of a languorous mood. Music is not only expressive of mood, but it also evokes moods. Carroll points out that the evocation of a mood can help in the recognition of the expressed mood in the music, as the evoked mood aids the discovery of meaning and movement in the music. The listener represents the movement of the music in imagination and bodily sensation. The evoked feelings of movement are associated with moods and this helps, on Carroll’s account, to appreciate a musical piece aesthetically.

## OBJECTIONS

### NO INTENTIONALITY, NO USE FOR MOODS

Zangwill (2004) argues that since a mood lacks an intentional object, it cannot have music as its intentional object and therefore it is not relevant to the aesthetic experience as such. The intentional object of a mental state is that which the state is about and directed toward. A mental state is intentional if it is directed at or represents something. An emotion is directed at an intentional object: if you are angry, you are always angry at someone or something. A mood such as melancholy, on the other hand, does not have a specific intentional object. It might be caused by a particular event, but it is not directed at anything. A breakup can cause a melancholic mood, but the melancholy itself functions as a background state which is directed at everything and nothing. Robinson accepts this analysis of mood, saying that “although it is the music that makes me feel sad, I’m not sad about the music” (Robinson, 2010, p. 667).

Zangwill argues that although music conveys moods, it does not follow that it is relevant to the aesthetic appreciation of music as such. He rightly assumes that everything that is relevant to the aesthetic experience of music, must have music as its intentional object, because otherwise it would not be an experience “of” music. Since a mood does not have an intentional object it cannot have music as its intentional object. Therefore, moods are not relevant to the aesthetic experience of music.

### MIND-WANDERING

Another objection against the aesthetic importance of musically evoked moods is formulated by Kivy (2006, 2007). While Robinson and Carroll maintain that music-induced moods help the listener to understand the expressive qualities of the music, Kivy considers moods irrelevant. He describes his view as enhanced formalism and argues that the expressivity of instrumental music is not felt, but recognized (Kivy, 2002). In other words, it is through our thoughts, not our moods, that we discover music’s expressivity. Instrumental music may be expressive of moods, but this expressivity is only recognized by attending to the formal aspects of the music. In order to recognize “*L’aprè*s-midi” as expressive of a languorous mood, the listener does not have to be in a languorous mood.

Kivy’s enhanced formalism presupposes that there is a canonical way of listening to music. This is the appreciation of the formal qualities of music, which represents the meaning of the music as it was meant to be. This canonical way of listening is free from personal associations. Music may induce moods and other kinds of personal associations, but they are not part of the appreciation of music as a fine art (Kivy, 2006, 2007). “*L’aprè*s-midi” always gets Lucy into a languorous mood, while if Jack listens to it, it brings back childhood memories which make him nostalgic. These personal associations and moods are highly subjective and are therefore not part of the aesthetic appreciation of music as such. The listener loses herself in mind-wandering, instead of focusing on the music.

## MUSICAL MOODS ARE CLUSTERS OF FEELINGS

That musical moods are aesthetically valuable is an intuition I share with Carroll and Robinson. However, if moods are not intentional it is indeed problematic to argue that moods are relevant to the aesthetic appreciation of music, but maybe moods can be conceived of as intentional after all. Michael Tye has developed such an account and claims that the philosophical orthodoxy on the topic of moods is wrong (Tye, 1995, pp. 93–131). Drawing on the work of neuroscientist Damasio (1994), Tye argues that we experience a mood if we feel a change in our body. Most of the time we are in a neutral state, but when we feel our bodies change in a certain way, we are experiencing a specific mood. So Tye offers a different understanding of mood: the felt character of mood consists of feelings, which have intentional objects. What it means for feelings to have intentional objects, is something I will address later on. The relation between music and feelings has been discussed, but this always remained separate from the debate on music and moods. My proposal is that if the experience of a mood is a cluster of feelings, we can introduce feelings to the debate on music and mood.

This provides a new account of musical moods, one which meets Kivy’s and Zangwill’s objections. The idea is that each mood is associated with a specific cluster of feelings. This does not mean that if you experience feelings X, Y, and Z it is necessarily implied that you have mood M. Every mood is different, and denominating moods is not a matter of logical inference. We usually find it hard to precisely describe every specific feeling we are experiencing. Nevertheless, if someone says that she in a “energetic” mood, we can get a sense of which feelings she might have. I will argue that music evokes certain feelings which can be associated with a specific mood. So I need to show that bodily feelings can be directed at the music and that clusters of these feelings are part of the aesthetic experience of the music.

Tye argues that bodily feelings have intentionality in the same way as emotions do (Tye, 1995, pp. 93–131). As I explained earlier, a mental state is intentional if it is directed at or represents something. For instance, the feeling of pain represents, according to Tye, a particular part of the body (i.e., the intentional object) as damaged. Each specific type of pain represent a specific type of damage: a little twinge of pain, for instance, represents a part of the body as being just mildly damaged. So for a mental state to represent the intentional object “as” something, means that a mental state is directed at an object and attributes information or content to it. In this sense feelings can be directed at intentional objects and represent them as something.

In order to explain what it means for a feeling to represent something in the context of music, it is helpful to look at a specific type of feeling which Damasio calls “background feelings” (Damasio, 2003, pp. 83–84), a notion inspired by philosopher Langer (1957) and developmental psychologist Stern (1985). Interestingly both Langer and Stern relate these feelings to music appreciation. Stern calls these feelings “vitality affects.” They are different from other feelings like the sensation of pain, pleasure, and hunger, in the sense that they represent dynamics. Vitality affects are intentionally directed at dynamic structures and they represent them as “surging, fading-away, fleeting, explosive, tentative, effortful, accelerating, decelerating, climaxing, bursting, and drawn out” (Stern, 1999, p. 68). In other words they represent actions in a specific way. When, for instance, an infant gets fed by her father, she not only perceives that her father is feeding her, but she also feels that when the spoon reaches her mouth the father moves his arm as if his action (i.e., the intentional object) is “climaxing.” Stern claims that infants can, long before they develop linguistic thought, can “represent these (dynamic experiences) in some non-verbal kinesthetic and bodily manner” (Stern, 2010, p. 69–70). This climaxing way of movement is not represented in thought, but is felt in the infant’s body, through her vitality affects.

Stern himself contends that these vitality affects occur while listening to music (Stern, 2010). Music has “dynamic aspects” such as crescendo, tension, and release. These dynamic aspects can be thought of in terms of vitality affects which represent them as “explosive,” “bursting,” or “drawn out.” Stern links the concept of vitality affects to Langer’s morphology of feeling (Stern, 2010, p. 37). Langer argues that music does not represent emotions as such, but rather the dynamics of feelings. Music is a symbolic representation of affective dynamics. “There are certain aspects of the so-called “inner life” – physical or mental which have formal properties similar to those of music – patterns of motion and rest, of tension and release, of agreement and disagreement, preparation, fulfillment excitation, sudden change, etc.” (Langer, 1957, p. 228) In order to describe the structural likeness of symbolical musical movements and the dynamics of our affective lives, Langer refers to psychologist Carroll Pratt’s famous dictum that music “merely sound[s] the way moods feel…” (Pratt, 1931, p. 203). As it are our vitality affects and not our ears that represent the musical dynamics of motion and tension as “explosive” or “bursting,” it might be better to say that music effectively *feels* the way moods feel.

Zangwill and Kivy rightly maintain that an entire mood cannot be relevant to the aesthetic experience of music as such. However, Tye claims that experienced moods consist of different feelings. Some of these feelings, I will argue, are not directed at the music while others may very well be part of the aesthetic experience. Music evokes vitality affects and a cluster of these vitality affects can be recognized as typical of a specific mood. That is what constitutes a “musical mood,” i.e., a cluster of vitality affects directed at the music. This argument is similar to Carroll’s, who claims that evoked bodily sensations of movement are associated with moods. I, however, think that it is important to distinguish between the “musical mood,” and the general mood that the listener is in.

This can be seen in the following example. Juliet attends a concert with her boyfriend, Romeo, and is in a very good mood. Juliet feels relaxed, in balance and senses a warm feeling of inner wellness. Some of these feelings are related to her bodily state, while other feelings are related to the presence of Romeo: the nice feeling of being on a the date and the loving feelings which are directed at Romeo. Also, the music itself may evoke certain vitality affects such as the feelings of “surging,” “fading-away,” and “fleeting.” These vitality affects, associated with a relaxed mood are part of the mood she is experiencing. In this case the cluster of vitality affects evoked by the music and the feelings she had before the concert are similar. Still, it would be wrong to say that Juliet’s entire mood is part of the aesthetic experience, since only the particular feelings which are directed at the music are.

Consider another case in which the general mood the listener is in and the musical mood are distinct. Before entering the music hall Juliet and Romeo have a heated argument. The music starts, but Juliet is still in an angry mood. The guilty look of Romeo annoys her and she is irritated by the tall man sitting in front of her. As Juliet feels a lot of tension in her body, she feels uncomfortable and has problems sitting still. Nevertheless, the music sounds very relaxing to her: the melody flows slowly and in a relaxed way to its climax and then fades away again. The music sounds harmonious, as if every note falls into place. The cluster of feelings that the music evokes is associated with a relaxed mood, so Juliet experiences the music as a relaxed mood, although her overall experience is one of mixed feelings. Some of the feelings she experiences are related to the angry mood she was in when the concert started. The other feelings, that the music evoked, are associated with a relaxed mood. In this case there is a difference between the musical mood, which is relaxed and Juliet’s initial angry mood. She experiences the music as relaxing, while her overall mood is an experience of mixed feelings.

This illustrates the idea that the listener’s entire mood is not relevant to aesthetic experience of music as such. However, musical moods are distinct from the listener’s general mood. Moods consist of different feelings and musical moods are clusters of music-induced feelings that are paradigmatic for a specific mood. The languorous mood of “*L’aprè*s-midi” consists of the cluster of feelings the music evokes, which are paradigmatic for a languorous mood. It is not the entire mood of the listener that is supposed to be languorous, but the music feels languorous nevertheless. Therefore I propose that the dictum “music feels the way moods feel” is a good way of describing the aesthetic relevance of moods.

## OBJECTIONS RECONSIDERED

Distinguishing between feelings associated with a mood and an entire mood provides a response to Zangwill’s and Kivy’s criticisms. Zangwill maintains that since moods lack intentional objects, the music you are listening to can never be the intentional object of the mood you are experiencing. However, the vitality affects are not simply caused by the music, they also represent its dynamics in a specific way. Thus, vitality affects have the dynamic aspects of the music as intentional objects, and therefore, the intentionality requirement is met and vitality affects can be part of the aesthetic experience as such.

Kivy argues that experiencing music-induced moods is mind-wandering; only attending to the formal qualities of music is essential to the canonical listening practice. Vitality affects, however, are a quick and dirty way of detecting the music’s dynamical aspects and represent expressive formal aspects of the music. If there is such a thing as a canonical listening practice, then these vitality affects are part of it and therefore Kivy’s criticism can be avoided. Kivy’s account is disembodied since he excludes everything that is felt in the body, while in my account the embodied experienced of music is essential for its aesthetic appreciation.

## CONCLUSION

The relation between mood and feeling has been overlooked in the literature on the aesthetic experience of music. Zangwill and Kivy rightly argue that the listener’s entire mood is not relevant to the aesthetic appraisal of music. I have contended that the experience of a mood consists of different feelings and some of these feelings are directed at the music, while others are not. The feelings directed at the music are vitality affects. Some clusters of these feelings can be recognized as typical feelings of a particular mood. Consequently we can say that music feels the way moods feel. These theoretical considerations show that the musical experience is not disembodied: the way music affects our bodies is an important aspect of aesthetic appreciation. I am hopeful that these theoretical considerations will inspire empirical research that explores further the relation between feeling, mood and music. Instead of focusing on which music is related to which mood, it might be fruitful to consider bodily feelings as mediators between mood and music.

## Conflict of Interest Statement

The author declares that the research was conducted in the absence of any commercial or financial relationships that could be construed as a potential conflict of interest.
